# Human colorectal cancer-derived carcinoma associated fibroblasts promote CD44-mediated adhesion of colorectal cancer cells to endothelial cells by secretion of HGF

**DOI:** 10.1186/s12935-019-0914-y

**Published:** 2019-07-24

**Authors:** Rongsheng Zhang, Fan Qi, Shengli Shao, Geng Li, Yongdong Feng

**Affiliations:** 1 Cancer Research Institute, Tongji Hospital, Tongji Medical College, Huazhong University of Science and Technology, Wuhan, 430030 Hubei China; 2 Department of Otolaryngology-Head and Neck Surgery Tongji Hospital, Tongji Medical College, Huazhong University of Science and Technology, Wuhan, 430030 Hubei China

**Keywords:** Colorectal cancer, Cancer associated fibroblasts, Adhesion, Metastasis

## Abstract

**Background:**

Carcinoma-associated fibroblasts (CAFs) are dominant components of tumor microenvironment, which has been reported to promote development, progression, and metastasis of cancer. However, the role of CAFs during adhesion process remains unknown. It has been hypothesized that CAFs contribute to adhesion to endothelial cells of colorectal cancer (CRC) via HGF/c-Met pathway.

**Methods:**

Clinical specimen and orthotopic liver metastasis model was used to investigate association between CD44 expression and propensity of metastasis in CRC. Human CRC derived cancer associated fibroblasts was isolated and its effect on migration and adhesion of CRC cells was investigated. We also confirm the conclusion on animal metastasis model.

**Results:**

In this study, clinical specimen and orthotopic liver metastatic model indicated that overexpression of CD44 is associated with CRC metastasis, and we found that colorectal cancer-derived CAFs (CC-CAFs) increased the adhesion and migration of CRC cells in vitro through up-regulation of CD44, we also found that CC-CAFs promoted adhesion and liver or lung metastasis in vivo. Mechanistically, we found that the expression of HGF increased tenfolds compared CC-CAFs with adjacent normal fibroblasts, and HGF promoted adhesion through up-regulation of CD44 via HGF/c-MET signal pathway.

**Conclusions:**

These results indicated that CC-CAFs-derived HGF induced up-regulation of CD44 which mediated adhesion of CRC cells to endothelial cells, and subsequently resulted in enhancement of metastasis of CRC cells, it could provide a novel therapeutic or preventive target.

## Background

Colorectal cancer is the second cause of cancer-related death, the prognosis is mainly dependent on the formation of metastasis site. Metastasis is a series of steps, including separation from primary tumor, invasion through surrounding tissue, entry of circulatory system, establishment and proliferation in distant location [1, 2]. CD44, a transmembrane protein may involve in the process, has been reported to be adhesion molecular and mediator of adhesion between tumor cells and endothelial cells [3] which could promotes subsequently metastasis, and has also been shown to be associated with bad prognosis in colorectal cancer [1, 4–7].

Carcinoma-associated fibroblasts which could include cancer-associated mesenchymal stem cells are the most abundant stromal cells in the tumor microenvironment. They promote tumor growth, angiogenesis and the malignant progression of various solid tumor through direct cell–cell contact or by paracrine secretion [8, 9], up-regulation of epidermal growth factor (EGF), interleukin-6 (IL-6), insulin-like growth factor, or hepatocyte growth factor in CAFs has been reported recent years [10–12]. Hepatocyte growth factor (HGF) is an important fibroblast secreted protein [13] which binds to Met, a specific receptor expressed in tumor cells and actives downstream signal that mediates development and progression of cancers [14, 15]. It has been reported that HGF/c-Met activation could subsequently result in up-regulation of CD44 expression [16], this implies that possible correlation exists between CAFs and adhesion process, however it has not been elucidated so far.

A comprehensive understanding of association between tumor microenvironment and metastasis is important to prevent tumor progress, in the present study, the clinical specimens and orthotopic liver metastatic model were used to identify gene that promote metastasis, then colorectal cancer-derived CAFs were isolated from primary human colorectal cancer tissue to investigate their effect on adhesion process and metastasis of colorectal cancer. We also investigated the underlying mechanisms which contribute to those effects.

## Materials and methods

### Ethics statement

Colorectal cancer tissues were obtained from patients who had undergone surgery at the Tongji Hospital, Huazhong University of Science and Technology. Written informed consent was obtained from all participants, and all procedures were authorized by the Ethical Committee of Tongji Hospital.

### Isolation of CAFs from colorectal cancer tissues and subsequent primary culture

Fresh colorectal adenocarcinoma tissues were obtained from eight patients who had undergone surgery, colorectal cancer-derived cancer associated fibroblasts (CC-CAFs) and primary colorectal normal fibroblasts (NFs) were isolated from colorectal cancer tissues and adjacent normal tissues respectively as our previous work [11]. Briefly, the specimens were soaked into 75% ethanol, then washed in phosphate buffered saline (PBS) with 1% penicillin/streptomycin. After mincing and digesting with collagenase IV (Invitrogen, CA, USA) for 3 h at 37 °C, PBS was used to wash the tissues twice, which were then passed through a 70 µm cell strainer (BD Falcon, CA, USA). The cells were centrifuged and cultivated in red blood cell lysis buffer to eliminate red blood cells, and then washed the remaining cells three times with PBS. Finally, the cells were disseminated on culture dishes. After the fourth passage, the human CC-CAFs were ready for use in subsequent experiments. For the co-culture experiment, CC-CAFs were grown on the 0.4 μm pore size transwell insert (Corning, USA) and the CRC cells were grown in the bottom well of the transwell chamber.

### Cell lines, reagents, transfections

Cell lines (LOVO, HUVEC, CT26 and SW48) were obtained from the American Type Culture Collection. The SW48, LOVO, and HUVEC were incubated in Dulbecco’s modified Eagle’s medium (DMEM) with high glucose content (Invitrogen, CA, USA) containing 10% fetal bovine serum (FBS; gibco, USA) and 1% penicillin/streptomycin, CT26 was maintained in RPMI1640 (Invitrogen, CA, USA) supplemented with 10% FBS and 1% penicillin/streptomycin. All cells were cultured in humidified atmosphere of 5% CO2 in air at 37 °C. SU11274 (Medchem express, USA), a specific chemical inhibitor of Met; Anti-HGF antibody (Sinobiological, China), a neutralizing antibody for HGF; recombinant human HGF (Peprotech, USA). GFP lentiviral particles were purchased from Genechem Co (china, shanghai). The transfection of lentiviral was conducted following instruction of manufacturer. For lentiviral transduction, 5000 cells/well were seeded on 96 well tissue culture plates and infected the following day with lentiviral particles (Santa-Cruz Biotechnology, Dallas, TX) at a MOI of 10 in the presence of 10 mg/ml polybrene, purchased from). GFP-expressing cells were selected with 2 μg/ml puromycin (Medchem express, USA) and enriched by three cycles of fluorescence-activated cell sorting (FACS).

### Flow cytometry staining and analysis

Cells were prepared as previously mentioned [11]. The labeled cells were analyzed on BD FACSverse (BD, Bioscience), and the data were processed by FlowJo software (Treestar).

### Collection of CC-CAFs condition medium

The condition medium was collected as our previously mentioned [11]. 2 × 106 CC-CAFs were seeded onto a 10-cm plate and cultivated with the complete medium. After 36 h, the medium was replaced with 5 ml of fresh F12/DMEM without serum and the cells were cultured for a further 24 h. The CC-CAFs culture medium (CC-CAFs CM) was collected and subjected to filter, stored it at − 80 °C.

### Patients and specimens preparation

The colorectal cancer primary tumor and CRC liver metastases were acquired from 10 patients who had undergone surgery. The isolated tissues were then fixed in 4% paraformaldehyde for 24 h, dehydrated in 25% saccharobiose solution overnight at 4 °C. After embedded in optimal cutting temperature (OCT) compound (Sakura Finetech, Tokyo, Japan), serial cryosections of 10 μm were cut and mounted on glass slides.

### Immunofluorescence assay

Immunofluorescence assay was carried out according to the standard protocols. The specific biomarkers of CAFs, α-SMA, FSP, vimentin and CD44 induced by the CAFs in SW48 and LOVO cells were determined by immunofluorescence assay. Cells planted on the glass slide and frozen sections were washed with PBS, following by fixing with 4% paraformaldehyde for 30 min and then permeabilized in 0.1% Triton X-100 for 25 min. After being blocked with goat sera at room temperature for 1 h, cells and sections were incubated with antibody against α-SMA (1:100; proteintech, China), vimentin (1:100; proteintech, China), FSP (1:100; proteintech, China) or CD44 (1:100; proteintech, China) overnight at 4 °C, rinsed with PBS, incubated with suitable Dylight 594 red-conjugated or Alexa Flour 488 green-conjugated secondary antibodies (1:500; Multi-Sciences Biotech Co. Ltd, Hangzhou, China) for 1 h at room temperature, washed with PBS thrice and costained with 10 μg/ml 40,60-diamidino-2-phenylindole (DAPI) (Sigma, USA) for 10 min and finally observed and imaged with an Olympus Fluoview 500 IX 71 confocal microscope (Tokyo, Japan). Images were digitally recorded at the same magnification and time of exposure.

### Static adhesion assay

Static adhesion assay was performed as previously described with modifications [1]. HUVECs were seeded onto 6-well plates at a density of 5 × 104 cells/well. Then LOVO or sw48 cells which stably transfected with GFP (pretreated with or without HGF or CC-CAFs CM) were plated (5 × 104 cells/well) and incubated for 30 min at 37 °C. The unattached cells were gently washed twice with 10% FBS-containing DMEM, the count of tumor cells adhered to HUVECs was examined under fluorescence microscopy. Then cells were digested with 0.25% trypsin, the SW48-GFP/LOVO-GFP cells and HUVECs were quantified by flow cytometry (BD, FACSVerse). The ratio of adhered sw48-GFP/LOVO-GFP cells to HUVECs were calculated as the ratio of sw48-GFP/LOVO-GFP cells to 5 × 104 cells.

### Quantitation of SDF-1 and HGF by enzyme-linked immunosorbent assay (ELISA)

Conditioned medium from the CC-CAFs was collected and HGF ELISA kit (eBioscience, CA, USA) was used to determine the concentration of HGF in CC-CAFs according to instruction of manufacturer. All tests were performed in duplicate. The plate was read at a wavelength of 450 nm. The concentration of HGF (pg/ml) was defined by making a standard curve with recombinant HGF. F12 without supplements served as the control. Expression of SDF-1 was investigated in a similar way using a human SDF-1 ELISA kit (eBioscience, CA, USA).

### Western blotting

Western blotting was carried out according to the standard protocols described previously [11]. We used primary antibodies raised against GAPDH (Santa Cruz Biotechnology, Biotechnology, CA, USA), Met, AKT, phospho-Met, phospho-AKT (Cell Signaling Technology, MA, USA), α-SMA, FSP and CD44. Goat anti-mouse and anti-rabbit antibodies conjugated with horseradish peroxidase were used as secondary antibodies (1:2000, Jackson ImmunoResearch, PA, USA). Protein bands were detected using enhanced chemiluminescence (ECL) reagents (Dura, Pierce, NJ, USA).

### Migration assay

We performed the migration of SW48 and LOVO cells using Transwell chambers (Corning, MA, USA) in a 24-well plate containing 8-µm pores as our previously mentioned [11]. Tumor cells in serum-free F12/DMEM were placed in the upper chambers and F12/DMEM containing 20% FBS was placed in the lower chamber. After 24 h, the cells that had migrated towards lower chamber were fixed with 4% paraformaldehyde for 10 min, then stained with crystal violet, and finally counted by bright-field microscopy.

### RNA extraction and real-time polymerase chain reaction (PCR) assays

Total RNA was extracted using TRIzol Reagent (Invitrogen, CA, USA) following the manufacturer’s protocol and was reverse-transcribed into complementary DNA (cDNA) using a Superscript Reverse Transcriptase Kit (Transgene, France). Super SYBR Green Kit (Transgen, France) was used to carry out real-time PCR in ABI7300 real time PCR system (Applied Biosystems). Following specific primer was used: GAPDH (5′-GAGAGACCCTCACTGCTG-3′ and 5′-GATGGTACATGACAAGGTGC-3′); vimentin (5′-AGTCCACTGAGTACCGGAGAC-3′ and 5′-CATTTCACGCATCTGGCGTTC-3′); fibroblast activation protein (FAP) (5′-ATGAGCTTCCTCGTCCAATTCA-3′ and 5′-AGACCACCAGAGAGCATATTTTG-3′); ACTA2 (5′-AAAAGACAGCTACGTGGGTGA-3′ and 5′-GCCATGTTCTATCGGGTACTTC-3′). PCR was performed according to instruction: 95 °C for 10 min; and 40 cycles of 95 °C for 15 s and 60 °C for 1 min. The relative gene expression levels for vimentin, FAP and ACTA2 were normalized against that of GAPDH in each sample, and each sample was run in triplicate and averaged.

### shRNA transductions and HGF treatment

For HGF, treatment cells were seeded at a concentration of 6 × 105 cells/well in a six well plate. After 24 h, the cell media was changed to serum-free media for an additional 24 h. Recombinant human HGF (PeproTech, USA) was then added to the wells at different concentration for 12 h. For shRNA transduction, CD44 shRNA and control shRNA expression lentiviral particles were purchased from Genechem Co (China, shanghai), lentiviral transduction was performed as mentioned previously.

### Orthotopic liver metastatic model

Animal assay were performed according to Wuhan Medical Experimental Animal Care Guidelines and were carried out in 6–10-week-old BALB/c mice bred in specific pathogen-free condition of the Animal Research Center of Tongji Medical College of Huazhong University of Science and Technology (Wuhan, China) approved by The Tongji Hospital of Huazhong University of Science and Technology. The liver metastasis model was based on orthotopic CRC model established previously with modification. Briefly, an abdominal midline incision was performed, the cecum exposed, and 5 × 105 CT26-GFP cells (in 50 μl Matrigel) were injected into the cecal wall. Then, the cecum was rinsed with distilled water to kill leaked tumor cells and repositioned into the abdomen. The abdomen and skin were closed using running suture. After 3 weeks, the mice were sacrificed, and the metastatic sites of liver were isolated. Then, Metastatic tumor fragments were minced into 1-mm cubes and digested in collagenase IV (Invitrogen, CA, USA) for 3 h at 37 °C, digested cells were washed twice with complete cell culture media and transferred into RPMI1640 (Invitrogen, CA, USA) containing 10% FBS and 1% penicillin/streptomycin and 1 μg/ml puromycin to eliminate cells except CT26-GFP cells derived from liver metastatic site, after selection process, the cells were analyzed by flow cytometry and western blotting.

### Animal experiment

We bred BALB/c (nu/nu) mice under specific pathogen-free (SPF) conditions of Animal Research Center of Tongji Medical College of Huazhong University of Science and Technology (Wuhan, China) approved by The Tongji Hospital of Huazhong University of Science and Technology. Mice were used for experiments at 6 weeks old. The animal experiment was carried out as described previously [11]. For in vivo lung metastasis model, sw48 cells with or without CC-CAFs were injected at a density of 1 × 106 in 100 μl PBS via the tail veins of the randomized mice (n = 7 per group). For intrasplenic injection of CRC cells, BALB/c (nu/nu) mice were anesthetized with pentobarbital sodium. A 1 cm cutaneous incision was made in the left flank and carried down through the peritoneal wall. The spleen was carefully exposed, and SW48 cells with or without CC-CAFs (5 × 106/100 μl) were injected under the spleen capsule. For short-term metastasis assay, 1 × 106 SW48-GFP cells were injected via tail vein, and 48 h later, pulmonary circulation perfusion was performed with PBS to eliminate circulating tumour cells, lungs were then taken out and fixed in 4% paraformaldehyde for frozen sections. The fluorescence cells per fields of lung sections were counted.

### Statistical analysis

SPSS 21.0 software (IBM Corp., Armonk, New York, USA) was applied for data analysis. All experiments were repeated 3 times in each group. The mean value of the measurement data was expressed as the mean and SEM. Comparisons among groups were by one-way analysis of variance (ANOVA), and multiple comparisons between the average number of samples were performed by LSD analysis. P < 0.05 indicated that the difference was statistically significant.

## Results

### Isolation of human colorectal cancer-derived cancer associated fibroblasts and immortalization

**Fig. 1:**
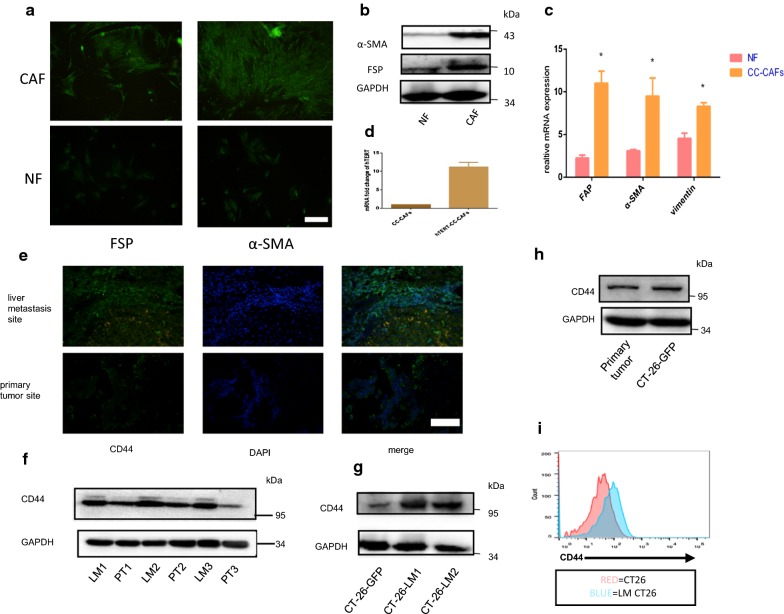
The characterization of human colorectal cancer-derived primary CAFs and NFs. **a** Immunofluorescence of FSP and α-SMA of CAFs and NFs (magnification, ×200). CC-CAFs were positive for the expression of FSP, α-SMA (**b**) the protein expression of CAFs and NFs was determined by western-blot. **c** The mRNA expression of FAP, α-SMA, vimentin was analyzed by Q-PCR, GAPDH serves as internal control. Error bars represent mean ± s.d; *P < 0.05, by unpaired two-sided Student’s t-test. **d** mRNA expression of hTERT was analyzed by Q-PCR, GAPDH serves as internal control (n = 3). **e** Expression of CD44 in liver metastasis site and primary tumor site (magnification, ×200). **f** The protein expression of liver metastasis site and primary tumor site was evaluated by western-blot (*LM* liver metastasis site, *PT* primary tumor site). **g**, **i** Liver metastasis site-derived CRC cells in orthotopic liver metastatic model was isolated, and expression of CD44 was determined by western-blot, and CD44 expression in GFP(+) subpopulation was analyzed by flow cytometry. **h** CD44 expression of primary tumor and CT26-GFP was evaluated by western blot and representative results from one of the three independent experiments are presented. Error bars represent mean ± s.d; *P < 0.05, **P < 0.01; ***P < 0.001; ****P < 0.0001, *n.s* not significant; by one-way analysis of variance (ANOVA)

The CC-CAFs and NFs were isolated from colorectal cancer tissue and adjacent normal tissue, respectively. Fibroblast specific protein (FSP), smooth muscle actin (α-SMA) were used to identify CC-CAFs. As shown in Fig. 1a, CC-CAFs were positive for the expression of FSP, α-SMA, Western blotting and real-time PCR confirmed that the expression of those markers in CC-CAFs were up-regulated compared with adjacent NFs (Fig. 1b, c). These results showed that we have isolated primary CC-CAFs and NFs successfully. The CC-CAFs was subsequently infected by hTERT expression lentiviral vector to produce immortalization, as shown in Fig. 1d, real-time PCR confirmed that mRNA expression of hTERT was increased after infection,primary CC-CAFs undergo aging after 8 generations, the CC-CAFs-hTERT can continue dividing even after 20 generations.

### CD44 was up-regulated in CRC metastases of clinical specimens and metastatic model-derived CRC cells

As shown in the Fig. 1e, a higher level of CD44 expression in CRC liver metastases compared with primary tumor was observed by immunofluorescence. The CD44 expression of liver metastasis site and primary tumor was further confirmed by western-blot, as shown in Fig. 1f, 3 pair of samples were evaluated which proved that up-regulation was not clinical sample-specific. To confirm correlation between liver metastatic propensity and expression of CD44, orthotopic liver metastatic model was established. In this study, mouse colon cancer cell line CT26 which engineered to express GFP and puro-resistance gene, the cell line was named CT26-GFP. CT26-GFP was used to injected into cecal wall, after 3 weeks, liver metastases were harvested, the cells were isolated and designated as CT26-LM (liver metastasis), and the cells was cultured in 5 μg/ml puromycin for 1 week to eliminate stroma cells. Western blotting showed the expression of CD44 in CT26-LM was up-regulated significantly compared with CT26 (Fig. 1g), the result was further conformed by flow cytometry analysis (Fig. 1i). to exclude possibility that in vivo environment might effect on expression of CD44, the expression of CD44 of primary tumor site in orthotopic liver metastatic model was evaluated by western-blot, as shown in Fig. 1h, similar CD44 expression can be observed compared primary tumor site with in vitro cultured CT26-GFP, taken together, those results indicated that CD44 expression was associated with metastatic propensity in CRC.

### CC-CAFs enhanced migration of CRC cells and CD44-dependent adhesion with HUVECs

**Fig. 2:**
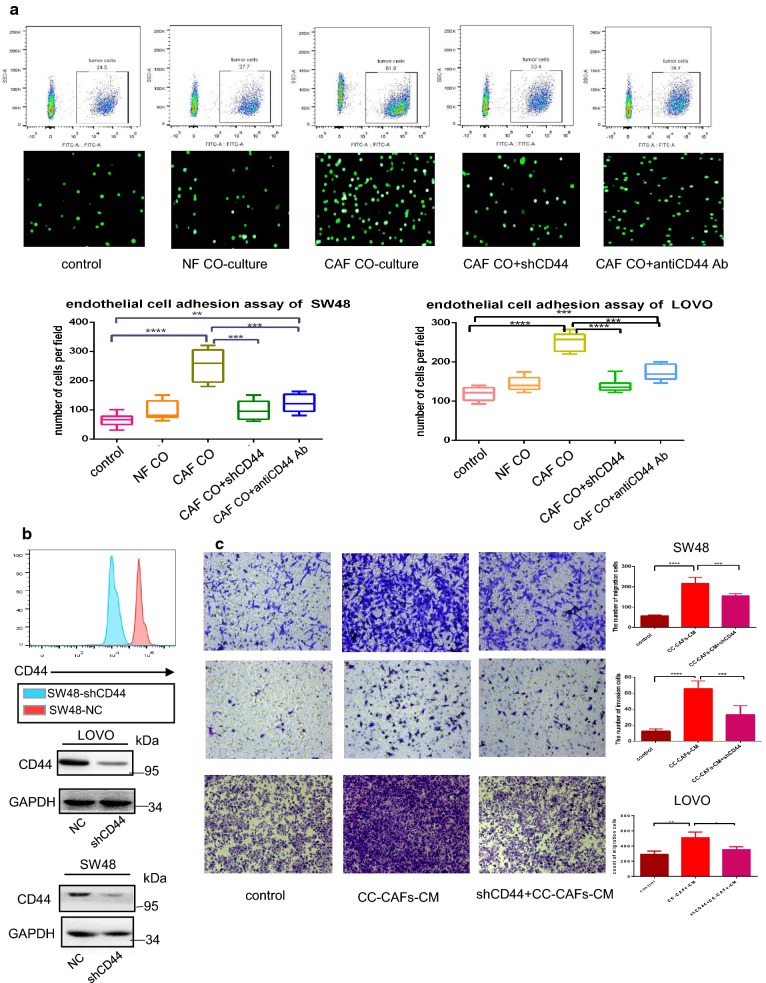
Human colorectal cancer-derived CAFs promoted CD44-dependent adhesion and migration in vitro. **a** The adhered SW48 were counted under fluorescence microscopy (bottom) and quantified by flow cytometry (top) (n = 6), adhered LOVO and SW48 was quantified in bottom. *P < 0.05, ***P < 0.001, ****P < 0.0001. **b** Expression of CD44 after transfection of CD44 shRNA was analyzed by western-blot and flow cytometry. **c** Transwell invasion (bottom) and migration assay (top) of SW48 cells and LOVO exposed to CC-CAFs-CM with or without transfection of CD44 shRNA (magnification, ×200). And migrated cells was quantified (n = 9), error bars represent mean ± s.d; **P < 0.01; ***P < 0.001; ****P<0.0001; by one-way analysis of variance (ANOVA)

To evaluate effect of CC-CAFs on adhesion of CRC cells, adhesion assay was performed. As shown in the Fig. 2a, after co-cultured with CC-CAFs, adhesion capacity of SW48-GFP cells or LOVO-GFP cells increased significantly, whereas such enhancement did not observed in CRC cells co-cultured with NFs. We then applied CD44 shRNA and anti-CD44 antibody to explore the role of CD44 in the adhesion induced by CC-CAFs. The effect of CD44 shRNA was shown in Fig. 2b, CRC cells adhesion induced by CC-CAFs was substantially reduced after shRNA transfection or anti-CD44 antibody treatment. To determine whether CC-CAFs have impact on the migration of CRC cells, the migration and invasion of tumor cells were evaluated in the presence or absence of CC-CAFs-CM, more migrated cells were observed when CRC cells were subjected to CC-CAFs-CM treatment. But the count of migrated cells was decreased after CD44 shRNA transfection (Fig. 2c).

### CC-CAFs derived HGF maintained adhesion and migration capacity of CRC cells through up-regulation of CD44

**Fig. 3:**
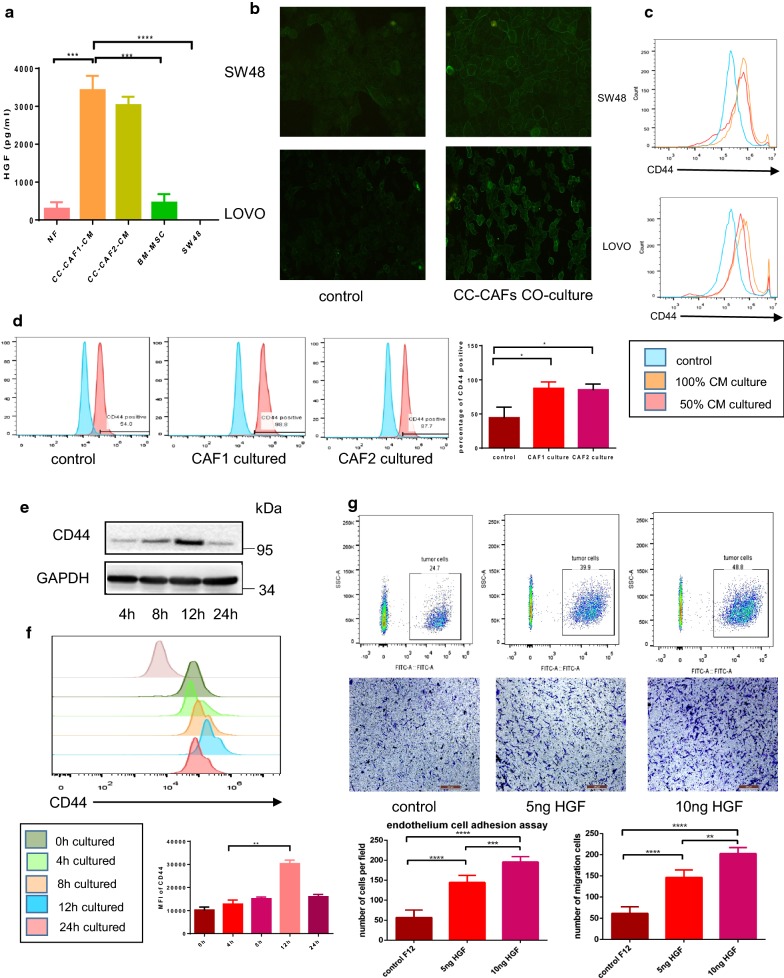
CC-CAFs-derived HGF maintained adhesion and migration capacity of CRC cells through up-regulation of CD44. **a** Quantitative analysis of HGF by using enzyme-linked immunosorbent assay (ELISA). The condition medium of NFs, SW48, BM-MSC and CC-CAFs were collected to detect level of HGF. The CC-CAFs were isolated from two patients (n = 3). Error bars represent mean ± s.d; ***P < 0.001; ****P < 0.0001. **b** Expression of CD44 in SW48 and LOVO cells with or without CC-CAFs-CM treatment was detected by immunofluorescence (magnification, ×200). **c** SW48 cells were cultured with different dilution ratio of CC-CAFs-CM for 12 h. CD44 expression was determined by flow cytometry in SW48 (top) and LOVO (bottom). **d** Expression of CD44 in SW48 co-cultured with or without 2 CAFs was evaluated by flow-cytometry, *P < 0.05. **e** SW48 cells were cultured with CC-CAFs-CM for different times. Protein expression was analyzed by western-blot. **f** SW48 cells were cultured with 5 ng/ml HGF for different times. CD44 expression was determined by flow cytometry. **P < 0.01. **g** Adhesion assay (top) and transwell migration assay (bottom) of SW48 cells subjected to different HGF concentration (magnification, ×100; scale bar: 250 μm). Error bars represent mean ± s.d; **P < 0.01; ***P < 0.001; ****P < 0.0001; by one-way analysis of variance (ANOVA)

**Fig. 4:**
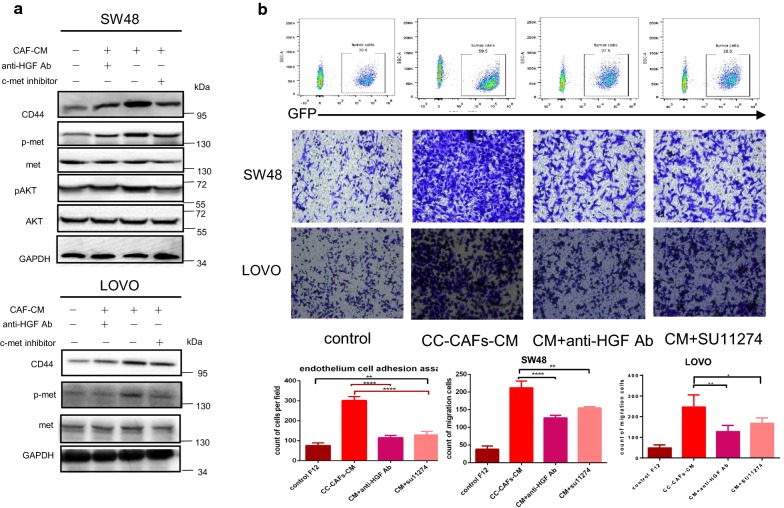
CC-CAFs-derived condition medium promotes migration and adhesion of CRC cells through HGF/MET/AKT signal in colorectal cancer. **a** Tumor cells were cultured in CC-CAFs-CM for 12 h with or without anti-HGF antibody or c-MET inhibitor SU11274. Protein expression was determined by western blotting and representative results from one of the three independent experiments are presented. **b** Adhesion assay (top) and migration assay (bottom) in SW48 cells and LOVO with or without anti-HGF antibody or c-MET inhibitor SU11274 (magnification, ×200). The migrated cells and adhered cells was quantified (n = 9). Error bars represent mean ± s.d; *P < 0.05, ***P < 0.001, ****P < 0.0001; by one-way analysis of variance (ANOVA)

To identify underling cytokine which promotes adhesion effect of CC-CAFs-CM, we cultured CC-CAFs or NFs in serum-starved F12 for 24 h, then determined the level of HGF using ELISA. As shown in the Fig. 3a, HGF in the CC-CAFs-CM was 3000 pg/ml which was about ten folds to supernatant of NFs or BM-MSC (bone marrow-derived MSC). A possible precursor of CC-CAFs. No HGF was detected in SW48, Interestingly, SDF-1 which has been widely reported existing in supernatant of CAFs did not detected in supernatant of CC-CAFs or NFs. CD44 expression of CRC cells was evaluated by immunofluorescence (Fig. 3b), a significantly up-regulation of CD44 was observed when CRC cells were treated by CC-CAFs-CM, the result was confirmed by flow cytometry analysis (Fig. 3c), and when 50% diluted CC-CAFs-CM was used to treat tumor cells, expression of CD44 was decreased compared with 100% CM which indicated that the CD44 up-regulation induced by CC-CAFs-CM was dose-dependent. To exclude possibility that this effect was clinical sample-specific, CAFs derived from 2 different patents was used to co-culture with CRC cells. As shown in Fig. 3d, CAFs from two patients all significantly increased CD44 expression of CRC cells. Time course experiment showed that CD44 expression on SW48 cells reached maximum levels (Fig. 3e) within 12 h of CC-CAFs-CM treatment, and the expression was reduced after 24 h of incubation. Similar time course can be observed when recombinant HGF was used to induce up-regulation of CD44 (Fig. 3f). To further confirm the role of HGF in the CC-CAFs-induced adhesion, recombinant HGF was used. Treatment with recombinant HGF enhanced adhesion or migration of CRC cells in dose-dependent manner (Fig. 3g), and when anti-HGF antibody was added to CC-CAFs-CM, significant reduction in the adhesion and migration of CRC cells can be observed (Fig. 4b).

Taken together, these data showed that the effect of the CC-CAFs on adhesion and migration of CRC cells was dependent on HGF secretion.

### CC-CAFs derived condition medium promotes up-regulation of CD44 through HGF/MET/AKT signal in colorectal cancer

Underling mechanism was investigated by western blotting. As shown in Fig. 4a, CC-CAFs-CM resulted in increased phosphorylation of MET, AKT and expression of CD44 in colorectal cancer cells. After the application of anti-HGF antibody, AKT, MET, activation and up-regulation of CD44 was partly inhibited, same result can be observed when c-MET inhibitor SU11274 was used, and the inhibitor blocked CC-CAFs-CM induced adhesion or migration significantly (Fig. 4b). These data indicated that CC-CAFs induced activation of HGF/MET/AKT result in up regulation of CD44 and subsequently adhesion or migration.

### CC-CAFs enhance adhesion and metastasis of CRC cells in vivo

**Fig. 5:**
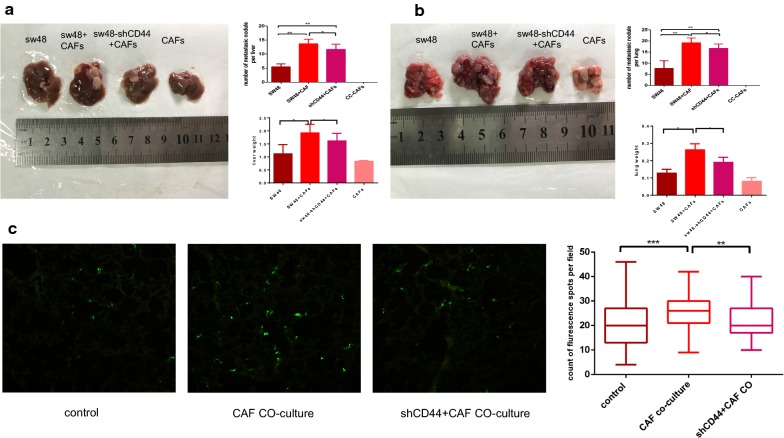
CC-CAFs enhanced adhesion and metastasis of CRC cells in vivo. **a** Liver tissue from BALB/c (nu/nu) mice of each group was obtained at 6 weeks, the number of liver metastatic foci was counted. And weight of liver was quantified. *P < 0.05, **P < 0.01. **b** Lung tissue from BALB/c (nu/nu) mice of each group was obtained at 8 weeks, the number of lung metastatic foci was counted. And weight of lung was quantified. *P < 0.05, **P < 0.01. **c** 1 × 10^6^ SW48-GFP cells were injected via tail vein, and 48 h later lungs were taken out for frozen sections. The count of sw48-GFP with or without transfection of shRNA CD44 or pre-co-cultured with CC-CAFs in frozen section per field was analyzed (magnification, ×100). Error bars represent mean ± s.d; **P < 0.01; ***P < 0.001; *n.s* not significant; by one-way analysis of variance (ANOVA)

To confirm the effect of CC-CAFs on tumor metastasis and the role of CD44 in vivo, we injected SW48 cells alone, CC-CAFs alone, SW48 cells mixed with CC-CAFs or SW48-shCD44 mixed with CC-CAFs via spleen capsule and tail vein. The mice were sacrificed 6–8 weeks later (n = 7). Compared with SW48 cells alone, co-injection of SW48 cells and CC-CAFs increased the number and size of tumor nodules in liver and lung and significantly enhance weight of lung and liver. But CC-CAFs-induced enhancement of metastasis was decreased when CD44 was knocked down, the injection of CC-CAFs alone did not result in formation of metastatic tumors (Fig. 5a, b). To conform the effect of CC-CAFs on adhesion of CRC cells in vivo, metastasis assay was performed. Compared with SW48-GFP, co-injection of SW48-GFP and CC-CAFs enhance the count of tumor cells adhered to endothelial cells of lung within 48 h significantly and knock down of CD44 can decrease the effect (Fig. 5c). These results demonstrated that CC-CAFs enhanced CD44-dependent adhesion and metastatic potential in vivo.

## Discussion

Solid tumor is comprising of cancer associated fibroblasts, macrophages, neutrophils, extracellular matrix, and tumor cells which close interact with stromal cells. Recent study reported that activated fibroblasts or MSC are present in tumor microenvironment and promote progression of cancer [17]. It have been reported that CAF actively secret cytokine such as SDF-1, IL6, IL8 which remodel tumor stroma and positive regulate cancer progression [18–21].

In the current study, orthotopic liver metastatic model and clinical specimen were used to figure out key molecular associated with metastasis, then CAFs was isolated from human colorectal cancer tissue and confirm CAFs phenotype. We observed that CC-CAFs enhanced the migration and adhesion with HUVEC of colorectal cancer cell in vitro, promoted metastasis of colorectal cancer in vivo, and determine the underling mechanism.

To begin with, CC-CAFs have an positive influence on migration and adhesion of CRC cells. The results showed that CO-culture with CC-CAFs enhanced migration and adhesion with HUVEC of CRC cells and accompany with up-regulation of CD44 expression, which was not observed in CO-culture with NFs, but this effect was abolished as administration of CD44 antibody or transfection of CD44 shRNA. Similar to our observation, Elliott et al. [1] reported that aggressive liver metastatic CRC cells line phenotype is associated with overexpression of CD44, and knock-down of CD44 impair CRC progression was also reported recent years [4, 5]. It implies that CC-CAFs potentially enhance colorectal cancer metastasis by up-regulation of CD44 whose association with metastasis has been observed in our clinical specimen and orthotopic liver metastatic model. We also investigated the underlying mechanism of pro-tumor effect of CC-CAFs, based on report by Benjamin et al. [22], HGF is a major component of cancer associated fibroblasts secretion, and have been showed to promote cancer progression [13, 23–25]. However, the mechanism has not decisively investigated in colorectal cancer. In the present study, higher level of HGF have been detected in CC-CAFs compared with BM-MSC or NFs, then, anti-HGF antibody and HGF were used to investigate role played by HGF in the effect. Anti-HGF antibody significantly attenuated migration and adhesion of CRC cells induced by CC-CAFs, up-regulation of CD44 and subsequent improvement of migration and adhesion were induced by HGF treatment. Similar to our findings, Yahata et al. [26] illustrated that up-regulation of CD44 and subsequently promotion of adhesion has been observed in breast cancer after HGF treatment, which suggested that HGF plays an important role in CC-CAFs induced metastasis of CRC.

We also found that activation of c-MET and AKT serves an important role in CC-CAFs induced metastasis of CRC, c-MET is receptor of HGF, and is expressed in epithelial cells [27, 28]. HGF induced activation of MET also triggers AKT or STAT3, induces epithelial–mesenchymal transition (EMT), and promotes multiple pro-tumor effects [29–33]. In the present study, activation of MET/AKT can be observed when CRC cells subjected to CC-CAFs derived condition medium culture, and this effect was partly decreased in the present of HGF antibody. Similar results can also be observed when c-MET inhibitor SU11274 was used, which also impaired CC-CAFs induced adhesion or migration.

We also investigated underling role played by CD44 in CC-CAFs induced metastasis and adhesion in vivo. CC-CAFs promoted liver and lung metastasis significantly, but the effect can be partly blocked after transfection of shRNA, which implied that CC-CAFs induced CD44 up-regulation may serves as pro-metastasis function. Based on report by Tomita et al. [34], short-term metastasis assay was performed, and similar results of tumor cell adhesion can be observed in 48 h, which imply CC-CAFs may promote CD44 expression and subsequently early metastasis site formation.

However, our work still has some limitations, the effect of CAFs on CRC cells was only evaluated using CAFs derived from 1 patient, we cannot exclude possibility that CAFs-promoted enhancement of adhesion of CRC cells might be clinical sample-specific. More investigation still need to be done.

## Conclusion

Our work proves for the first time that Human colorectal cancer-derived carcinoma associated fibroblasts (CC-CAFs) have an influence on adhesion which involved in the processes of metastasis of colorectal cancer and confirmed its effect on migration of CRC cells. HGF expression in CC-CAFs was higher that of normal fibroblasts, CC-CAFs promote metastasis of colorectal cancer through HGF/c-Met which induce up-regulation of CD44 whose correlation with metastasis can be observed in clinical specimen and orthotopic liver metastatic model. In brief, our results showed that CC-CAFs promoted metastasis of colorectal cancer by up-regulation of CD44 through HGF/MET/AKT signal. And provide a possible therapeutic strategy for CRC.

## Data Availability

Not applicable.

## Abbreviations

CRC: colorectal cancer
CAF: cancer associated fibroblasts
HGF: hepatocyte growth factor
